# Endobronchial lipoma a rare cause of pleural empyema: a case report

**DOI:** 10.4076/1757-1626-2-6377

**Published:** 2009-07-21

**Authors:** Yassine Ouadnouni, Mohammed Bouchikh, Salma Bekarsabein, Abdellah Achir, Mohammed Smahi, Yassine Msougar, Najat Mahassini, Abdellatif Benosman

**Affiliations:** 1 Department of Thoracic Surgery, Ibn Sina University HospitalRabatMorocco; 2 Department of Anatomical Pathology, Ibn Sina University HospitalRabatMorocco

## Abstract

Benign neoplasm of the endobronchial tree is quite rare, while endobronchial lipoma is extremely rare. The irreversible pulmonary damage is due to progressive bronchial obstruction; even so, pleural empyema is exceptionally encountered in a case of endobronchial lipoma. We report a case of a 47-year-old man who had left lung pneumonia with hemoptysis. The chest computed tomography showed cystic bronchiectasis with pleural effusion, Flexible bronchoscopy revealed a round tumor on the left main bronchus.

## Introduction

Endobronchial lipoma is an uncommon benign tumor, 127 cases have been reported till now. The late diagnosis of benign neoplasms can lead to irreversible pulmonary damage. We report the case of endobronchial lipoma complicated by chronic cystic bronchiectasis and pleural empyema.

## Case presentation

**Figure 1.**
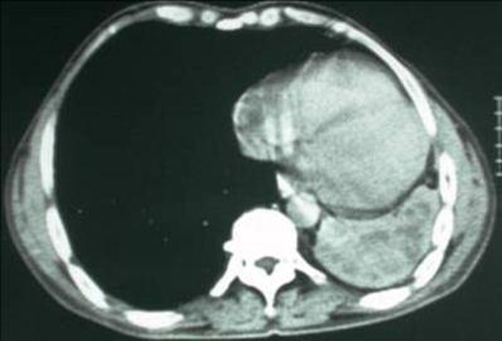
Computed tomography (CT) scan showing multiple cystic cavities in the left lung.

**Figure 2.**
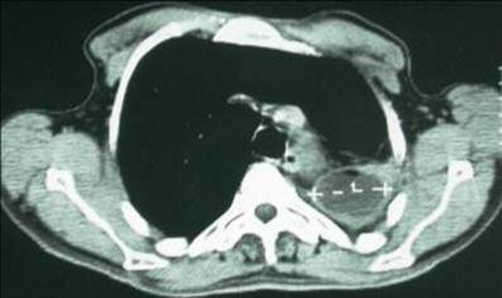
Computed tomography (CT) scan showing pleural pocket.

**Figure 3.**
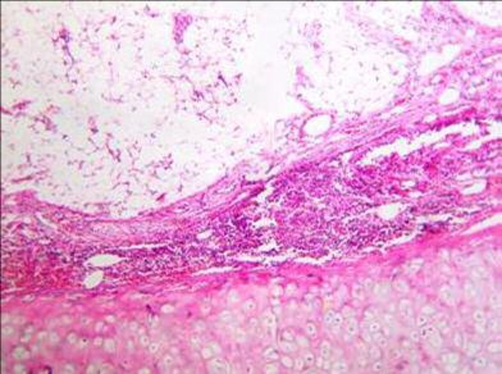
Photomicrograph showing mature fat cells surrounded by respiratory epithelium and the submucosa contained a moderate number of chronic inflammatory cells (hematoxylin-eosin stain x10).

A 46-year-old man African origin and Moroccan nationality who had been treated for a pulmonary tuberculosis in his childhood. He was a heavy smoker (25 pack-years). The patient suffered for many years from chronic bronchitis and chronic left-side pneumonia, treated each time with amoxicillin-clavulanic acid. He was admitted in the emergency department with a temperature of 39°C, sputum production, hemoptysis and pleuritic chest pain. During the clinical examination, the patient was tachypneic at rest; auscultation revealed decreased breathing sounds at the left lung’s base. Blood tests showed an elevated white cell count (18500 cells/μL). Chest radiograph showed atelectasis of the left lung and pleural effusion. A chest computed tomography (CT) scan showed multiple cystic cavities in the left lung, the volume within was diminished with encysted pleurisy (Figures 1, 2). Flexible bronchoscopy revealed a round tumor completely filling the lumen of the left main bronchus, and the biopsy of the neoplasm revealed lipidic cells. Several cytobacteriologic examinations of the sputum did not reveal any malignant cells or Koch’s bacillus, and culture of the sputum turned out to be negative. Preoperatively, the diagnosis of endobronchial lipoma associated to chronic cystic bronchiectasis complicated by pleural effusion was suggested. A left posterolateral thoracotomy was performed; a pleural pocket was opened, bringing out a purulent fluid. Cutting the left main bronchus revealed a smooth and yellow tumor. Because of the irreversible damage to the left lung, a pneumonectomy was performed. Macroscopic examination of the resected specimen showed a sessile mass measuring 1.5 cm, attached to the bronchial mucosa, and cystic bronchiectasis. Microscopically, the endobronchial tumor mainly contains a mature fatty tissue surrounded by respiratory epithelium; whereas the submucosa contains a moderate number of chronic inflammatory cells. Consequently, the tumor was diagnosed as an endobronchial lipoma (Figure 3). In the pleural fluid, a Streptococcus pneumoniae was detected; it proved to be sensitive to amoxicillin-clavulanic acid. The patient had an uncomplicated postoperative course, he was discharged 9 days after the operation, and was asymptomatic after 36-month follow-up.

## Discussion

Lipoma is a benign mesenchymal neoplasm of fat which is most common in the subcutis. In the usual type, it looks like mature fat, surrounded by a delicate capsule. It is extremely rare in the bronchus. The fat cells were located in the peribronchial and occasionally the submucosal tissue of large bronchus, with a reported incidence between only 0.1 and 0.5% in all lung tumors. The tumors are more frequent in middle-age men; some authors claim that smoking and obesity are significant risk factors for endobronchial lipoma [1]. Most cases reported in the literature, emphasize that the tumors occurred on the right side, and the most frequent were located in the first three subdivisions. In the present case, endobronchial lipoma was located in the left main bronchus and seal off the lumen completely.

Endobronchial lipomas produce a round or oval mass with smooth-surface, yellowish, and covered by respiratory epithelium. The tumor causes respiratory symptoms due to partial or total obstruction of bronchus and secondary lung destruction. Common symptoms include a persistent cough, sputum production, dyspnea, chest pain, recurrent fever and pneumonia. Hemoptysis is uncommon, related to the avascular nature of lipomas, but can occur as a result of postobstructive infection [2].

For asymptomatic patients, the chest radiography hardly shows obvious signs of intrabronchial mass (enlarged hilar). However, it exhibits clear indirect signs (parenchymatous consolidation, bronchiectasis). Other less frequent radiography observed a pleural effusion. Our patient presented atelectasis of the left lung and pleural effusion; a bacteriologic examination has detected the presence of Streptococcus pneumoniae. Pleural empyema associated to the endobronchial lipoma was only recorded in three cases, and this is the fourth English-language case reported [1].

In 1982, Sommer et al reported the first case of a lipoma identified by CT, the tumor is often a homogeneous mass with fat density (from −70 HU to −140 HU) and no enhancing contrast [3].

The diagnosis may be suggested by endoscopic aspects, but bronchoscopic biopsy frequently does not confirm it. Simmers et al reported that chronic obstructive pneumonia may induce sufficient nuclear atypia to suggest malignancy in endobronchial brush cytology of this tumor [4].

The exhaustive review by Muraoka et al shows that a correct preoperative diagnosis is possible only in 31% of 64 patients and that a thoracotomy is mandatory in 74% of patients, pulmonary resection was performed for 36 patients [2]. Surgical treatment, including pulmonary resection, has been often indicated: first, when there still a doubt about the diagnosis even with all the techniques, then, when a malignant tumor or a peripheral lung destruction is associated.

These tumors can be removed by bronchoscopy; in 1981, Dumon et al reported the first case of endoscopic resection for endobronchial lipoma [5].

## Conclusions

Endobronchial lipoma is a rare entity that can cause irreversible damages to lung parenchyma, unless the diagnosis and treatment are carried out in time. The conservative method such as bronchoscopic removal is recommended, otherwise, surgery is an alternative option for the undetermined benign or parenchyma destruction.

## Abbreviations

CT: Computed tomography
HU: Hounsfield unit
